# Modification of Ramie Fiber via Impregnation with Low Viscosity Bio-Polyurethane Resins Derived from Lignin

**DOI:** 10.3390/polym14112165

**Published:** 2022-05-26

**Authors:** Muhammad Adly Rahandi Lubis, Sucia Okta Handika, Rita Kartika Sari, Apri Heri Iswanto, Petar Antov, Lubos Kristak, Seng Hua Lee, Antonio Pizzi

**Affiliations:** 1 Research Center for Biomass and Bioproducts, National Research and Innovation Agency, Cibinong 16911, Indonesia; 2 Research Collaboration Center for Biomass and Biorefinery between BRIN and Universitas Padjadjaran, National Research and Innovation Agency, Cibinong 16911, Indonesia; 3 Department of Forest Products, Faculty of Forestry and Environment, IPB University, Bogor 16680, Indonesia; sucia_okta@apps.ipb.ac.id; 4 Department of Forest Product, Faculty of Forestry, Universitas Sumatera Utara, Medan 20155, Indonesia; 5 JATI—Sumatran Forestry Analysis Study Center, Universitas Sumatera Utara, Medan 20155, Indonesia; 6 Faculty of Forest Industry, University of Forestry, 1797 Sofia, Bulgaria; p.antov@ltu.bg; 7 Faculty of Wood Sciences and Technology, Technical University in Zvolen, 96001 Zvolen, Slovakia; 8 Laboratory of Biopolymer and Derivatives, Institute of Tropical Forestry and Forest Product, Universiti Putra Malaysia, Serdang 43400, Malaysia; lee_seng@upm.edu.my; 9 LERMAB-ENSTIB, University of Lorraine, 88000 Epinal, France; antonio.pizzi@univ-lorraine.fr

**Keywords:** bio-polyurethane resins, impregnation, lignin, ramie fibers, thermal stability, mechanical properties

## Abstract

The purpose of this study was to prepare low-viscosity lignin-based polyurethane (LPU) resins for the modification of ramie (*Boehmeria nivea* (L.) Gaudich) fiber via impregnation to improve the fiber’s thermal and mechanical properties. Low-viscosity LPU resins were prepared by dissolving lignin in 20% NaOH and then adding polymeric 4,4-methane diphenyl diisocyanate (pMDI, 31% NCO) with a mole ratio of 0.3 NCO/OH. Ramie fiber was impregnated with LPU in a vacuum chamber equipped with a two-stage vacuum pump. Several techniques such as Fourier-transform infrared (FTIR) spectroscopy, differential scanning calorimetry, thermogravimetric analysis, pyrolysis-gas chromatography–mass spectroscopy, field emission-scanning electron microscopy coupled with energy dispersive X-ray (EDX), and a universal testing machine were used to characterize lignin, LPU, and ramie fiber. The LPU resins had low viscosity ranging from 77 to 317 mPa·s^−1^. According to FTIR and EDX analysis, urethane bonds were formed during the synthesis of LPU resins and after impregnation into ramie fibers. After impregnation, the reaction between the LPU’s urethane group and the hydroxy group of ramie fiber increased thermal stability by an average of 6% and mechanical properties by an average of 100% compared to the untreated ramie fiber. The highest thermal stability and tensile strength were obtained at ramie impregnated with LPU-ethyl acetate for 30 min, with a residual weight of 22% and tensile strength of 648.7 MPa. This study showed that impregnation with LPU resins can enhance the thermal and mechanical properties of fibers and increase their wider industrial utilization in value-added applications.

## 1. Introduction

Currently, lignin is mostly obtained as waste and by-product of the pulp and paper industry, with an estimated annual volume of 50–70 million tonnes, and only around 2% of the extracted lignin is commercialized for various purposes [1]. Lignin is one of the major organic components in black liquor from the kraft pulping process, estimated to be 35–46% of the total dry solids [2]. Isolated lignin, also known as technical lignin, is a mixture of various lignin fractions that show differences in monolignol units, bond patterns, and different molecular weights [3]. As a result of this situation, each fraction has its own set of physical and chemical properties. Lignin fractionation is carried out to obtain a more uniform lignin structure, resulting in added value for lignin applications by producing specific polymer properties [4].

Lignin fractionation was carried out by extraction method using organic solvents, such as ethyl acetate, methanol, ethanol, acetone, dioxane, toluene, and chloroform. The principle of choosing a solvent is based on the partial solubility of lignin at different polarity levels [3]. The fractionated lignin showed different chemical and thermal characteristics by using organic solvents on industrial lignin [4,5]. Lignin is an abundant natural polymer that can strengthen bonds and can be decomposed in nature, so lignin is expected to be used as a sustainable, bio-based alternative feedstock to synthetic polymer materials. Due to its availability, low cost, and versatile chemical properties, commercial utilization of lignin in value-added products has gained a significant industrial interest as a green material for the replacement of fossil-derived products in various industrial applications, such as adhesives, polyesters, carbon fibers, bioplastics, and as polyols in the production of polyurethanes [6,7,8].

Polyurethane (PU) is a polymer composed of urethane bonds (NH–(C=O)–O) synthesized between isocyanate (NCO) and alcohol (OH) groups [9]. The process of synthesis and cross-link formation occurs when one monomer contains at least two isocyanates (diisocyanates), and the other monomer contains two alcohols (diols or polyols) [10]. The structure and properties of PUs are influenced by the type of diisocyanate, polyol, and the synthesis process [11]. Polyols used in the synthesis of PUs are generally petroleum-derived polyols. The rising environmental concerns associated with the production of sustainable products from renewable natural resources in order to lessen human reliance on diminishing fossil reserves necessitates the search for alternative bio-based feedstocks as a polyol in polyurethane synthesis [12]. Many studies have been conducted using bio-polyols derived from renewable and environmentally friendly natural resources. Alternative raw materials for bio-polyols in PU synthesis can be in the form of carbohydrate-based materials, natural phenolic compounds [13,14,15], vegetable oils, and lignocellulosic biomass [12,16,17], and today, bio-PU derived from renewable materials without the usage of isocyanates has also been created using the same biosourced ingredients [18,19,20,21]. PUs are used in various fields, such as automotive, building and construction, packaging, and biomedical products [22]. The use of PU has also increased as an impregnation material in ramie fiber to improve its thermal and mechanical properties [15,23].

Among the biopolyol resources, lignin has been extensively studied due to its availability, aromatic nature, and hydroxyl functionality [24]. Lignin has been explored as raw material for making polyurethanes due to the presence of free phenolic, aliphatic, carboxylic, and hydroxyl groups, which are able to interact with isocyanate [25,26]. Also the NCO content of lignin bio-PU decreases compared to the unmodified PU batch, which leads to efficient crosslinking and the presence of less monomer in the final system [15]. Two main ways of incorporating lignin in synthesis of PU are either direct incorporation of technical lignin without modification [27,28] or incorporation of modified lignin via esterification, etherification reactions, and depolymerization processes [29]. Varied PU materials with lignin as sole polyol resulted in brittle and hard polymers ascribing the intrinsic stiffness of lignin and low molecular mobility of diisocyanates, therefore soft composites are mostly incorporated as co-polyols to enhance the ductility of the resulting PU polymer [30]. The modified lignin exhibits high solubility in water and good compatibility between lignin and PU, contributing to the enhanced hydrogen bonding and superior mechanical properties of resultant PU composite [31].

In addition to increasing the bio-based content in the PU composites the use of lignin or modified lignin with polyurethane material improves enhanced crosslinking density, biodegradability, hydrophobicity, antioxidant properties, flame retardancy, mechanical strength, thermal stability, delamination, abrasion resistance, and UV-blocking ability [32,33,34]. The effects of synthesis parameters on the polymerization process, such as temperature, type of the polyol, type of isocyanate, type and amount of lignin, and NCO/OH ratio were intensively studied [34,35]. Lignin-based PU (LPU) prepared from low- and medium-molecular-weight lignin fractions are characterized by high modulus of elasticity and high tensile strength.

Ramie fiber has a better tensile strength value than cotton, hemp, and flax fibers [36]. Impregnation of ramie fiber is aimed at increasing the fiber’s thermal stability and fire resistance as a functional raw material [23]. Ramie fiber has low flame retardancy because it contains 80–90% cellulose and hemicellulose [37]. Therefore, chemical treatments are needed to increase the fiber resistance properties and enhance its thermal stability. Some refractory additives used are nitrogen, halogen, and phosphorus-based compounds [38]. Fiber impregnation is one of the modification methods carried out by filling the fiber using chemicals to improve certain desired properties. In this study, fractionated lignin using ethyl acetate and ethanol as a polyol was used in LPU development, and was applied as an impregnation material to modified ramie fiber to improve its specific properties, i.e., thermal stability and mechanical properties. The analyses performed included functional group analysis using Fourier-transform infrared (FTIR) spectroscopy, chemical analysis using pyrolysis-gas chromatography-mass spectroscopy (Py-GCMS), analysis of thermal properties using differential scanning calorimetry (DSC), thermal stability analysis by thermogravimetric analysis (TGA), field emission-scanning electron microscopy (FE-SEM) coupled with energy dispersive X-ray (EDX), and evaluation of mechanical properties (tensile strength and modulus of elasticity) of ramie fiber using a universal testing machine (UTM).

## 2. Materials and Methods

### 2.1. Materials

Materials used in this study were black liquor from the kraft pulping process from *Acacia mangium* wood (Tanjung Enim Lestari Pulp and Paper Company, Muara Enim, Indonesia). The black liquor had the following characteristics: solids content 76.8, moisture content 27.8%, and pH—12.1. Standardized kraft lignin from Sigma Aldrich (CAS No. 8068-05-1) was used as a standard. Chemicals used for isolation and fractionation of lignin were hydrochloric acid (HCl 1M, analytical grade, Merck, Darmstadt, Germany), sulfuric acid (H_2_SO_4_ 72%, analytical grade, Merck, Germany), ethyl acetate (analytical grade, Merck, Darmstadt, Germany), ethanol (ethanol 97%, analytical grade, Merck, Darmstadt, Germany), dioxane (1,4-dioxane, analytical grade, Merck, Darmstadt, Germany), sodium hydroxide (NaOH 20%), and distilled water. Polymeric 4,4-methane diphenyl diisocyanate (pMDI, 31% NCO content) was purchased from Anugerah Raya Kencana Company, Tangerang, Indonesia to prepare the Bio-PU resins. Degummed ramie fiber (*Boehmeria nivea* (L.) Gaudich) was supplied from Rabersa Company, Wonosobo, Indonesia.

### 2.2. Isolation and Fractionation of Lignin

Lignin was isolated by a single-step precipitation method based on the published method [39]. Acid precipitation was done by adding hydrochloric acid (HCl 1M) into a mixture of black liquor and distilled water until the solution reached pH 2. The solution was separated from the lignin precipitate with a pipette, washed three times with distilled water, then stored in the freezer for 24 h. After that, the lignin precipitate was vacuum filtered out of the solution and dried in a 45 °C oven for 24 h. The yield of lignin was calculated by dividing the weight of isolated lignin and black liquor.

Fractionated lignin was obtained using a single-step fractionation method with modification with two different solvents such as ethyl acetate (EtAc) and ethanol (EtOH) [40]. Lignin was mixed with EtAc and EtOH with a ratio of 1:15 in a 500-mL beaker glass. The fractionation process was carried out at room temperature for 24 h with a 200-rpm stirring speed. The soluble and insoluble fractions were separated using a Buchner funnel and filter paper. A rotary evaporator (Rotavapor^®^ R-300, Buchi, Flawil, Switzerland) condensed the dissolved fraction. The lignin samples were also dried for 24 h at 45 °C. The lignin fractions were used as a pre-polymer of LPU resins.

### 2.3. Characterization of Lignin

Various analytical methods were used to determine the moisture content, ash content, purity level, and total phenolic hydroxyl groups of lignin. Briefly, the moisture content (MC) was evaluated by drying samples in an oven at 105 °C for 24 h [41]. The ash content of lignin was evaluated by heating the sample for 6 h at 525 °C in a furnace [42]. The purity of isolated lignin was determined using a standard method [43,44]. A vial bottle was filled with around 0.3 g of lignin. After that, 3 mL of 72% H_2_SO_4_ was added and mixed for 2 h at 150 rpm with a magnetic stirrer. The blank solution was prepared with 3 mL of H_2_SO_4_ 72% and 84 mL of distilled water. In addition, the sample was autoclaved for 1 h at 121 °C. The solution was filtered using an IG3 filter glass (CTE33, IWAKI, Shizuoka, Japan). The residue was dried in the oven at 105 °C for 24 h. A UV–Vis spectrophotometer (UV-1800, Shimadzu, Japan) was used to measure the absorbance of the filtrate at a wavelength of 240 nm. The Equations (1)–(3) were used to determine acid-soluble lignin (ASL), acid-insoluble residue (AIR), and acid-insoluble lignin (AIL).

(1) $$ASL\;\left(\%\right)=\frac{UVabs\times volume\;filtrate\times dilution}{\varepsilon \times A\times cuvette\;length}\times 100\%$$

(2) $$AIL\;\left(\%\right)=AIR\left(\%\right)-Ash\;content\;\left(\%\right)$$

(3) $$Acid\;Insoluble\;Residue\;\left(AIR\right)\;\left(\%\right)=\frac{C-B}{A}\times 100\%$$

Description:

*ε* = absorptivity constant of biomass at specific wavelength (L/g cm)

*A* = weight of sample without moisture content (g)

*B* = dry weight of IG3 filter glass (g)

*C* = dry weight of IG3 filter glass and AIL (g)

The total phenolic hydroxyl groups in lignin were measured according to a published method [45]. The sample was prepared by dissolving 20 mg of lignin in 10 mL of dioxane and 10 mL of NaOH 0.2 M. To eliminate insoluble particles, the solution was filtered using a 0.45 µm nylon filter. In addition, 50 mL of solvents were used to dilute 4 mL of the original solution. NaOH 0.2 M, pH 6 buffer, and pH 12 buffer were all utilized as solvents. The solution was diluted until it reached a final concentration of 0.08 g L^−1^. UV measurements were taken in the 200–600 nm range using a UV–Vis spectrophotometer with a pH 6 buffer solution as a reference. The maximum absorption occurred at the absorbance of 300 nm and 350 nm, used for calculations. The total phenolic hydroxyl group was calculated using Equation (4) [45].

(4) $$\mathrm{Total}\;\mathrm{OH}\;(\mathrm{mmol}\;g^{-1})=\left((0.425\times A300\mathrm{nm}\;\left(\mathrm{NaOH}\right)+0.182\times A350\mathrm{nm}\;\left(\mathrm{NaOH}\right)\right)\times a$$

Description:

*A* = absorbance

*a* = correction term (L g^−1^ cm^−1^) = 1/(c × 1) × 10/17

*c* = concentration of lignin solution (g L^−1^)

Functional groups of isolated and fractionated lignin were examined using FTIR spectroscopy (SpectrumTwo, Perkin Elmer Inc., Hopkinton, MA, USA) with the universal attenuated total reflectance (UATR) method. The average accumulation was recorded as 16 scans with a resolution of 4 cm^−1^ and wavelengths from 4000–400 cm^−1^ at a temperature of 23 ± 2 °C.

A DSC instrument (DSC 4000, Perkin Elmer, Hopkinton, MA, USA) was used to examine the thermal characteristics of isolated and fractionated lignin. The samples were heated at a rate of 10 °C min^−1^ in a nitrogen atmosphere with a 20 mL min^−1^ flow rate at temperatures ranging from −50 to 350 °C without annealing. Each sample’s endothermic and exothermic peak temperature (*Tp*) and transition glass (*Tg*) temperature were calculated automatically using Pyris 11 software (Version 11.1.1.0492, Pyris, Hopkinton, MA, USA). The *Tp* and *Tg* values was measured by creating a baseline from the onset and the end temperature of the thermogram.

Thermal stability of isolated and fractionated lignin was investigated using TGA instrument (TGA 8000, Perkin Elmer, Hopkinton, MA, USA). A conventional ceramic crucible was filled with around 20 mg of sample and heated in a nitrogen atmosphere at a 20 mL min^−1^ flow rate. The heating temperature varies from 25 to 750 °C, with a 10 °C min^−1^ heating rate. Percentages of weight loss, weight loss rate, and residue were calculated using Pyris software (Version 11, Pyris, Washington, MA, USA).

Py-GCMS (QP 2020NX, Shimadzu, Kyoto Japan) was used to examine the lignin components. Lignin samples of 500–600 μg were put into the SF PYI-EC50F eco-cup, covered with glass wool, and Py-GCMS analyzed the samples. The eco-cup was pyrolyzed for 0.1 min at 500 °C utilizing multishot pyrolysis (EGA/PY-3030D) linked to a GC/MS QP-2020 NX system (Shimadzu, Japan) with an SH-Rxi-5Sil column MS with a film thickness of 30 mm × 0.25 mm id 0.25 µm, 70 eV electrons, and helium as a carrier gas. The pressure utilized was 20.0 kPa (15.9 mL min^−1^, 0.61 mL min^−1^ column flow). The temperature profile for GC was 50 °C for 1 min, then raised to 280 °C at a rate of 5 °C min^−1^ and maintained at 280 °C for 13 min. Pyrolysis products were detected by comparing the retention time and mass spectrum data in the 2017 NIST LIBRARY program.

### 2.4. Preparation of Bio-Based PU Resins

Two LPU resins were prepared using fractionated lignin, namely L-EtAc and L-EtOH, and pMDI at 0.3 NCO/OH mole ratio. The fractionated lignin was dissolved in a 1:10 (*w/v*) solution of NaOH 20% and treated with a pMDI solution in acetone (8% *w/v*). For the polymerization procedure, the mixture was mechanically stirred for 30 min at a speed of 500 rpm. The viscosity of LPU resins was measured using a rotational rheometer (RheolabQC, AntonPaar, Graz, Austria) under a constant shear of 100/s at 25 °C.

### 2.5. Evaluation of LPU Resins Properties

Similar to lignin, the LPU resins were also characterized for their properties. FTIR spectroscopy (SpectrumTwo, Perkin Elmer Inc., Hopkinton, MA, USA) coupled with the UATR method was used to investigate the functional groups of Bio-PU resins. Thermal properties of LPU resins were investigated using DSC (DSC4000, Perkin Elmer, Hopkinton, MA, USA) at a heating rate of 10 °C/min under a nitrogen atmosphere with a 20 mL min^−1^ flow rate at temperatures ranging from −50 to 350 °C. The endothermic and exothermic peak temperature (*Tp*) and transition glass (*Tg*) temperature were calculated automatically using Pyris 11 software (Version 11.1.1.0492, Pyris, Washington, MA, USA). The *Tp* and *Tg* values was measured by creating a baseline from the onset and the end temperature of the thermogram. Furthermore, thermal stability of LPU resins was investigated using a TGA instrument (TGA 4000, Perkin Elmer, Hopkinton, MA, USA). A conventional ceramic crucible was filled with around 20 mg of sample and heated in a nitrogen atmosphere at a 20 mL min^−1^ flow rate. The heating temperature varies from 25 to 750 °C, with a 10 °C min^−1^ heating rate. Percentages of weight loss, weight loss rate, and residue were calculated using Pyris software (Version 11, Pyris, Washinton, MA, USA).

### 2.6. Impregnation of Ramie Fiber with LPU Resins

Ramie fibers were impregnated in a 1 L vacuum chamber with a two-stage vacuum pump (VC0918SS, VacuumChambers.ue., Białystok, Poland) according to the published work [23]. Approximately 5 g of ramie fiber were immersed in 50 mL LPU resins and the mixture underwent vacuum impregnation for 30, 60, and 90 min at 25 ± 2 °C under 50 kPa pressure. The impregnated fiber was then dried for 24 h in a 60 °C oven and stored in a ziplock before testing.

### 2.7. Examination of Ramie Fiber Properties

The fiber diameter was measured from 10 fibers for each type of ramie fiber. The measurement was performed using a light microscope (BX63, Olympus, Tokyo, Japan) coupled with imaging software (Labspec 6, Horiba, Kyoto, Japan).

The weight gain of ramie fiber was calculated by dividing the mass of ramie fibers after impregnation by the initial mass of ramie fibers [15]. The change in functional groups of original and impregnated ramie fibers were examined using FTIR spectroscopy (SpectrumTwo, Perkin Elmer Inc., Hopkinton, MA, USA) coupled with the UATR method similar to lignin and LPU resins.

DSC (DSC4000, Perkin Elmer, Hopkinton, MA, USA) was used to examine the thermal properties of original and impregnated ramie fiber. The samples were heated at a rate of 10 °C min^−1^ in a nitrogen atmosphere with a 20 mL min^−1^ flow rate at temperatures ranging from −50 to 350 °C. Each sample’s endothermic and exothermic peak temperature (*Tp*) was calculated automatically using Pyris 11 software (Version 11.1.1.0492, Pyris, Washington, MA, USA).

TGA (TGA 4000, Perkin Elmer, Hopkinton, MA, USA) was used to investigate the thermal stability of original and impregnated ramie fibers. A conventional ceramic crucible was filled with around 20 mg of sample and heated in a nitrogen atmosphere at a 20 mL min^−1^ flow rate. The heating temperature varies from 25 to 750 °C, with a 10 °C min^−1^ heating rate. Percentages of weight loss, weight loss rate, and residue were calculated using Pyris software (Version 11, Pyris, Washington, MA, USA).

Surface morphology and nitrogen content of ramie fibers before and after impregnation were investigated by scanning a bundle of ramie fibers using FE-SEM (Quattro S, Thermo Fisher Scientific, Waltham, MA, USA) coupled with an EDX (Ultim Max, Oxford Instrument, Abingdon, UK) at a magnification of 500 times, using 3.0 kV of accelerated voltage, and a Ka1 X-ray source. The FE-SEM detector was the Everhart–Thornley detector (ETD), which is a secondary electron and back-scattered electron detector. The presence of nitrogen in ramie fibers indicated that the LPU resins has impregnated and bonded with the fibers via urethane bonds (R_1_–NH–(C=O)–O–R_2_).

Mechanical properties of untreated and impregnated ramie fibers, i.e., tensile strength and modulus of elasticity were evaluated based on ASTM D3379-75 using an universal testing machine (AG-X series, Shimadzu, Kyoto, Japan) [46]. A single fiber separated by strand bonds was employed as the specimen. The specimen length varied from 20 mm to 30 mm, and the total fiber length was almost three times than that of the specimen. The specimens were evaluated at a temperature of 23 °C with a load cell of 5 kN.

## 3. Results

### 3.1. Properties of Isolated and Fractionated Lignin

The lignin yield in this study was 35.9%, which was within the expected range of 20–40% [1]. The main characteristics of isolated lignin are presented in Table 1. The MC of lignin was 5.1%, whereas the ash content was 0.3%. The lignin MC was found to be lower than in earlier research works, when the lignin ash content ranged from 0.5% to 4.3% [47,48]. The AIL and ASL were used to determine lignin purity. The lignin isolate had an AIL content of 82.5% and an ASL value of 12.8%, respectively. Washing during the isolation process is expected to eliminate contaminants and other compounds besides lignin, resulting in a higher total lignin yield. The ash content of the isolated lignin in this study was discovered to be low (<1%), indicating that the precipitation and purification process used in this study was carried out correctly.

The fractionation yields are given in Table 2. The L-EtAc and L-EtOH had remarkably different fractionation yields. The yield of L-EtAc was 28.9%, which was lower than the yield of L-EtOH, which was 73.5%. The yield of L-EtOH was determined based on prior research that found the maximum solubility of fractionated technical lignin in a 70–80% ethanol solvent [3]. In organic solvents, where the ester product (EtAc) has low lignin solubility, the difference in yield between L-EtAc and L-EtOH is determined by lignin solubility. In addition, the type of lignin, aliphatic hydroxyl number, and molecular weight also influence lignin solubility in organic solvents [49]. According to the UV method’s results, the total phenolic OH groups of L-Standard, L-Isolated, L-EtAc, and L-EtOH ranged from 7.2–8.1%. Due to the obvious kraft pulping process, the phenolic OH content in L-isolated has a condensed chemical structure [40]. The result showed that L-EtAc had a higher phenolic hydroxyl group compared to L-isolated and L-EtOH, and a similar number to the L-standard.

**Figure 1:**
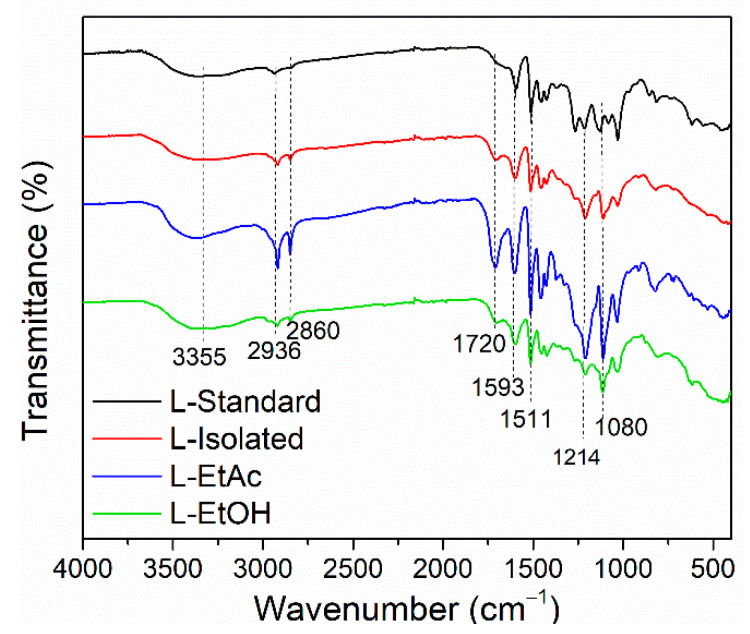
FTIR spectra of lignin and fractionated lignin.

The results of the FTIR analysis indicated that L-Isolated, L-EtAc, and L-EtOH had relatively the same absorption band as L-Standard, demonstrating that lignin isolation and fractionation did not affect the lignin structure (Figure 1 and Table 3). The presence of O–H stretching vibrations in phenolic O–H and aliphatic O–H groups caused the absorption band at wavenumber 3400–300 cm^−1^ to suggest broad-wave absorption. The C–H stretching in the CH_2_ and CH_3_ groups, part of the common material for lignocelluloses, is strongly connected to the spectrum at wavenumber 2930–2840 cm^−1^. L-EtAc had the maximum intensity at wavenumbers 2917 cm^−1^ and 2849 cm^−1^ [50,51]. C=O stretching was detected in the unconjugated ketone and aldehyde group at wavenumbers 1712–1702 cm^−1^, with L-EtAc having the maximum intensity at 1708 cm^−1^. Aromatic C=C stretching of the aromatic ring of lignin has a wavenumber of 1610–1590 cm^−1^, and the C=C of aromatic skeletal vibrations of the phenyl-propane (C9) skeleton has a wavenumber of 1595–1510 cm^−1^ [52,53].

The aromatic skeleton vibrations relate to the spectra generated at wavenumbers 1400 cm^−1^ and 1700 cm^−1^. Because of the isolation and fractionation procedures used, L-Isolated, L-EtAc, and L-EtOH did not change the aromatic structure of lignin. The wavenumber of 1470–1460 cm^−1^ indicated C–H deformation (asymmetric) on methyl, methylene, and methoxyl groups. C–H deformations in CH_2_ and CH_3_ groups and C-H aromatic ring vibrations at 1460–1420 cm^−1^ were determined. The C–O–C of guaiacyl ring (G-units) (phenolic groups) and C–O–C of aromatic acetyl groups were defined in the wavenumber 1222–1200 cm^−1^. Wavenumber 1115–1110 cm^−1^ indicated the existence of Ar-CH deformation (syringol), C–O stretching, and C=O stretching (G), with the most incredible intensity at L-EtAc. In addition, the spectra showed the C=O(H) and C=O(C) from aliphatic O–H and ether groups, as well as aromatic C-H deformation from guaicyl at wavenumbers 1044–1030 cm^−1^ [50,51]. The C–O stretching in guaiacyl was characterized as a peak at 1265 cm^–1^. The peak at 1080 cm^−1^ indicated the aliphatic O–H group, and ether had a higher intensity in the L-Standard, which was not detected in the L-Isolated, L- EtAc, or L-EtOH. The L-standard employed was guaiacyl lignin extracted from softwood. Softwood lignin has a higher intensity at wavenumber 1269 cm^−1^ than hardwood lignin and a lower intensity at wavenumber 1030 cm^−1^ [15,54].

**Figure 2:**
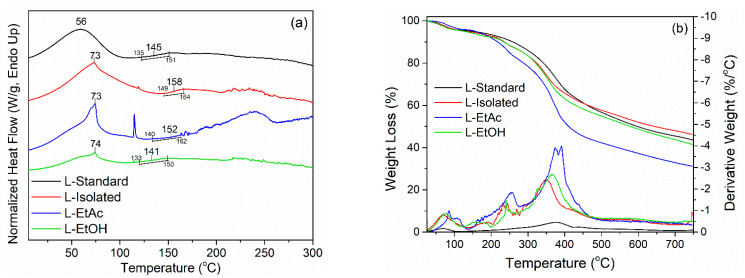
Thermal characteristics of lignin (**a**) DSC, (**b**) TGA-DTG.

DSC was used to evaluate the thermal properties of lignin. The glass transition temperature (Tg) of lignin or modified lignin samples is most commonly determined using DSC [51]. A graphical representation of the DSC analysis results for L-Standard, L-Isolated, L-EtAc, and L-EtOH is shown in Figure 2a. The results indicated that when lignin is heated, it undergoes two endothermic processes. The first endothermic reaction (Tp_1_) shows how lignin releases water. The Tp_1_ was found at temperatures below 100 °C, specifically at 56 °C for L-Standard. Meanwhile, L-Isolated and L-EtAc were formed at 73 °C, and L-EtOH was formed at 74 °C. The second endothermic process, also known as the glass transition temperature (Tg), occurs whenever the lignin structure changes, potentially reducing rigidity. Because of the polymer’s complex structure, Tg lignin is sometimes challenging to detect. However, the range of variations in the curve can occasionally be seen [55,56]. The Tg values derived from L-Standard, L-Isolation, L-EtAc, and L-EtOH ranged from 141–158 °C, reflecting the Tg for lignin, which was generally between 100–180 °C [51]. Because of the wide range of Tg, there are variances in the flexibility and stiffness of lignin at high temperatures, which will influence its use as a bio-polyol.

The thermal stability of isolated and fractionated lignin was determined by TGA-DTG analysis. When lignin is heat-treated at temperatures up from 25 to 750 °C, three stages of lignin degradation is apparent (Figure 2b). Evaporation of water contained in lignin occurs in the first step at temperatures ranging from 50 °C to 100 °C. Evaporation peaks at 70–80 °C, with a derivative weight of 0.8–1% °C^−1^. Furthermore, at a temperature of 150–250 °C, there was a weight loss due to carbohydrate decomposition, with a derivative weight of 2% °C^−1^ [15]. There was a substantial decrease in lignin weight after reaching 300 °C, particularly in L-EtAc. Degradation of the lignin polymer and the release of CO and CO_2_ chains in the lignin structure begins at a temperature of 300–400 °C; additional decomposition of the aromatic ring of lignin occurs at a temperature of 500 °C [8]. With a derivative weight of 2.5% °C^−1^, the biggest weight loss peak occurred at 350 °C in L-Isolated and L-EtOH. Meanwhile, at higher temperatures, L-EtAc demonstrated the highest weight loss, specifically at 390 °C, with a higher derivative weight of almost 4% °C^−1^. The L-Isolated had the highest combustion residue of 46.25%, followed by L-Standard with 43.51%, L-EtOH with 41.47%, and L-EtAc with 31.22%. Because it may improve the aromatic polymer content of LPU, lignin represents an excellent alternative as a substitute for conventional polyols in the production of LPU resins. Furthermore, due to its aromatic structure and cross-links, lignin is appropriate for utilization at high temperatures [56].

Py-GCMS was used to determine the lignin composition of isolated and fractionated lignin. Lignin is a polymer composed of three hydroxyl alcohols with varying degrees of methoxylation: p-coumaryl alcohol, coniferyl alcohol, and sinapyl alcohol. Each monolignol produces three types of lignin units: P-hydroxyphenyl (H), guaiacyl (G), and syringol (S). According to the Py-GCMS data, L-EtAc had more syringol groups than L-Isolated and L-EtOH (Table 4). L-Isolated had a S/G ratio of 0.96, L-EtAc had a ratio of 1.13, and L-EtOH had a ratio of 0.99. The chemical reactivity of various technical lignins is influenced by the S/G ratio. Previous studies revealed that the S/G ratio in lignin can change greatly depending on the method, bleaching procedure, and bioethanol generation at the time of pulping [57].

### 3.2. Characterizations of Lignin-Based Bio-PU Resins

**Figure 3:**
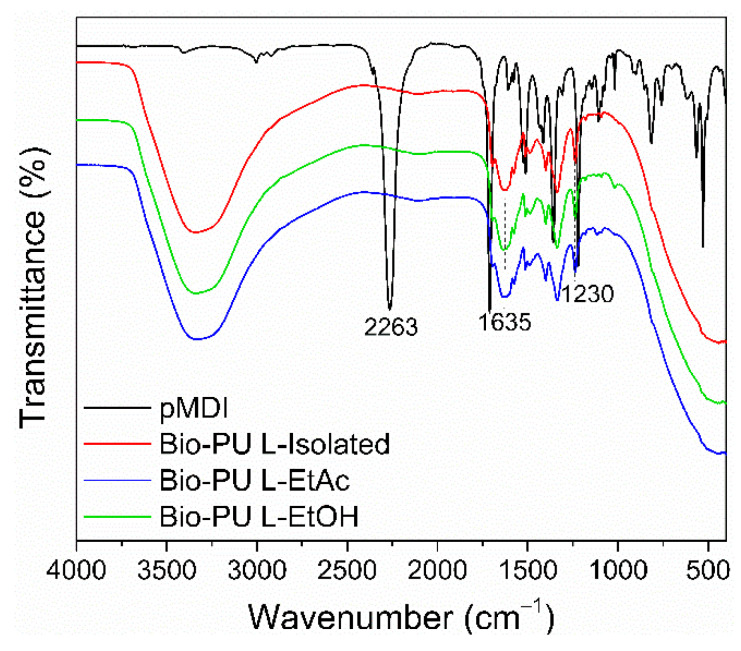
FTIR spectrum of lignin-based Bio-PU.

In this study, L-Isolated, L-EtAc, and L-EtOH were used as prepolymer to prepare LPU resins. Even though the isocyanate group has an electropositive carbon, it interacts rapidly with the hydroxyl group in lignin, forming a urethane bond. The development of functional groups in LPU resins derived from L-EtAc and L-EtOH was detected using FTIR (Figure 3). The C=O stretching vibration is influenced by hydrogen bonds between hard segments and dipole–dipole interactions between carbonyl groups [58]. The presence of an isocyanate group (–N=C=O) was shown by a strong peak at wave number 2263 cm^−1^, which is a characteristic of pMDI. The C=O stretching of aliphatic ketone and carboxylic acid was shown in the pMDI spectrum at a wavenumber of 1710 cm^−1^, aromatic C–C groups were visible at the peak of 1530 cm^−1^, and 1524–1511 cm^−1^ indicated the presence of N–O stretching on pMDI [59].

There was no peak at wavenumber 2263 cm^−1^ in LPU resins. It is possible to assume that the NCO group in pMDI interacted seamlessly with the OH group on lignin to create a urethane group and cross-links in LPU resins. At wavenumbers 3600–3200 cm^−1^, the stretching vibration of the O–H group may be seen in a wide range. The additional hydroxyl, which does not react with the isocyanate, might result from the interaction between lignin and NaOH, utilized as a solvent in this study. In LPU resins, a typical peak at wavenumber 1635 cm^−1^ indicated the structure of C=O group from the urethane bond. A substituted mono amide and C-H bending were formed with a wavenumber of 1553–1500 cm^−1^. A cyclohexane N–H group has a wavenumber of 1341 cm^−1^, while the C–N group has a wavenumber of 1250–1020 cm^−1^ [58,60]. This spectrum illustrates how polyols such L-Isolated, L-EtAc, and L-EtOH may be employed to produce LPU resins via the formation of urethane linkages with pMDI.

**Figure 4:**
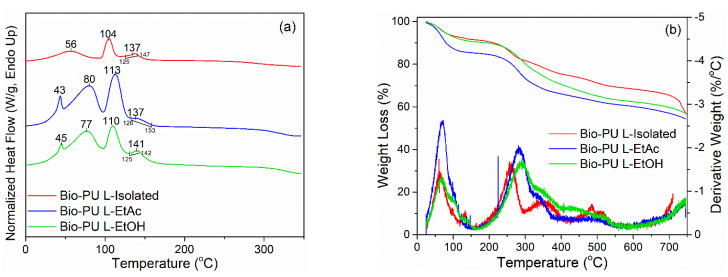
Thermal characteristics of lignin-based bio-PU: (**a**) DSC, (**b**) TGADTG.

Thermal characteristics of LPU resins were determined using DSC analysis. The thermogram of DSC obtained on LPU resins is shown in Figure 4a. During the heating of LPU, an endothermic peak was formed. It occurred because of the LPU resins requires additional heat energy to flow into the sample to increase the temperature. The curve area shows the amount of energy involved or heats absorbed in an endothermic reaction, and this factor indicates the development of bonds in the molecular structure system. *Tp1* of LPU is around 43–80 °C, indicating the beginning of water evaporation in LPU, and *Tp2* is about 104–113 °C, indicating the evaporation during the polymerization process [61]. Meanwhile, the *Tg* was found to be around 137–141 °C. The *Tg* is one of the thermal characteristics parameters that represents the polymer transition at a specific temperature [62]. The *Tg* value indicates that urethane production has resulted in forming a hard segment in LPU. The higher the *Tg* of LPU, the more formation of urethane linkages from the reaction of –OH of lignin and –NCO of pMDI.

Thermal stability of the LPU resins was examined by TGA analysis. The thermogram of LPU resins is displayed in Figure 4b. At a temperature of 80–100 °C, the weight loss of LPU resins reached 1.5% °C^−1^ for L-Isolated and L-EtOH; by contrast, L-EtAc had a higher weight loss of 2.5% °C^−1^. During polymerization, the initial weight loss is due to the evaporation of water and compounds in the unreacted lignin [23]. At 200–300 °C, the urethane bond decomposes in three phases [63]. The dissociation of the urethane and carbon dioxide linkages and the evaporation of isocyanates are generally the first steps in the deterioration of LPU [60]. At a temperature of 350 °C, oxidative decomposition of the urethane group occurs, resulting in the formation of primary compounds, alkenes, and carbon dioxide, with a relatively small average weight loss. The aromatic ring of lignin and the primary oxidative degradation of lignin, and the formation of secondary amines from LPU are decomposed around 450–600 °C [8,63].

In this study, the LPU weight loss percentage was not more than 50%, ranging from 43.0–45.6%. The LPU derived L-EtOH had the lowest weight loss of 43.0% and residue of 57.0%, followed by L-Isolated with 43.5% and 56.5% of weight loss and residue, respectively. The most significant weight loss was determined for the L-EtAc at 45.6%. The obtained weight loss of rigid or semi-rigid PU resins was around 60–75% [64], indicating that the LPU in this study had better thermal stability. Thermal stability refers to both thermal and heat resistance. High thermal stability polymers generally have a higher melting point, less thermal softening and decomposition, less weight loss during high-temperature heating, and a higher heat deflection temperature under load [63].

### 3.3. Characteristics of Ramie Fibers

**Figure 5:**
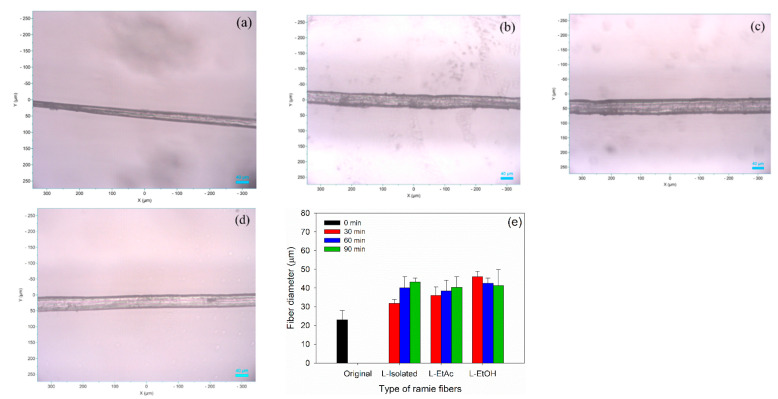
Typical microscopy analysis of ramie fibers before and after impregnation at 10× magnification: (**a**) original ramie fibers, (**b**) ramie–L-isolated—90 min, (**c**) ramie–L-EtAc—90 min, (**d**) ramie–L-EtOH—90 min, (**e**) average diameter of ramie fiber.

Typical microscope images of ramie fiber before and after impregnation are displayed in Figure 5a–d. The fiber is relatively narrow in diameter, regular, and without segmentation. These characteristics are comparable with the typical feature of ramie fiber [65]. The alteration in the average fiber diameter can be determined before and after impregnation (Figure 5e). The average fiber diameter of original ramie fiber was 22.5 μm. The fiber diameter generally became larger after impregnation, showing that the fiber diameter increased with the increase of impregnation time. The average fiber diameter of ramie after impregnation of 90 min could reach up to 46.1 μm, where impregnation with LPU-L-isolated produced greater fiber diameter compared to LPU derived from L-EtAc and L-EtOH. Longer impregnation time provided more time to the LPU resins entering the fiber, therefore swelled the fiber and increased the diameter. Except for impregnation with LPU-L-EtOH, the fiber diameter decreased with the increased impregnation time. This might be attributed to that longer impregnation time provided more time for EtOH in the LPU resins to evaporate rather than impregnating the fiber.

The weight gain (WG) value was used to calculate the rate of LPU resins penetration into ramie fibers during the impregnation process. The WG of impregnated ramie fiber as a function of the type of LPU resins and the impregnation time is presented in Table 5. The WG of the ramie fiber varied depending on the type of LPU resins. There was a significant influence on the fiber’s WG with different impregnation times of 30–90 min. Ramie impregnated with LPU L-Isolated had a higher WG than Ramie LPU L-EtAc and ramie LPU L-EtOH-impregnated fibers. The WG of ramie LPU L-Isolated was 12.4−15.6%, ramie LPU L-EtAc was 8.7−10.9%, and ramie LPU L-EtOH was 6.2−7.5%. Differences in the viscosity of the LPU resins generated by each lignin influence the difference in WG of each LPU. The LPU L-isolated had the lowest viscosity of 77.0 mPa s^−1^, while the bio-PU L-EtAc, and L-EtOH had higher viscosity of 206.6 mPa s^−1^ and 316.9 mPa s^−1^, respectively. This condition could make the LPU L-isolated penetrated the ramie fiber more efficiently. The fiber diameter of ramie fibers is much wider than cotton, hemp, and flax fibers, with a range of 40–60 µm [36]. This condition makes it easier for the LPU resins to penetrate the ramie fiber and increase the WG of ramie fibers after impregnation [15]. Furthermore, increased impregnation time was significantly correlated with the WG of ramie fiber.

**Figure 6:**
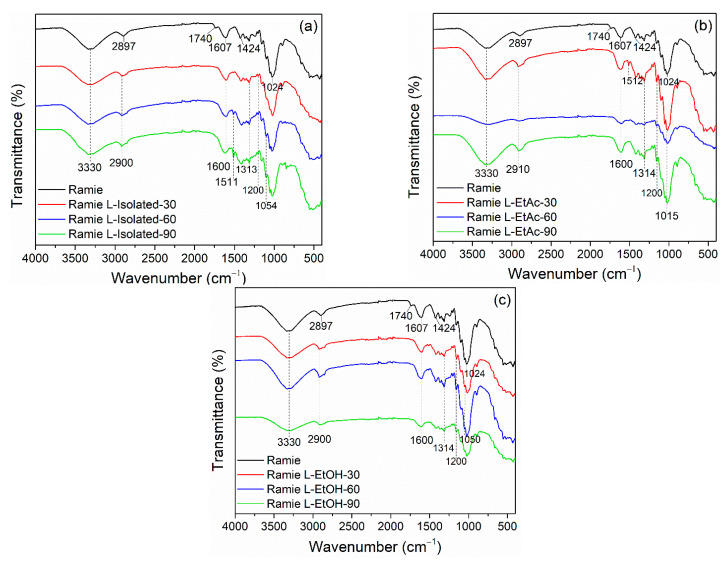
FTIR spectra of ramie fiber impregnated with lignin-based bio-PU at different times: (**a**) ramie impregnated with L-isolated, (**b**) ramie impregnated with L-EtAc, (**c**) ramie impregnated with L-EtOH.

The fiber impregnation aims to enhance the fiber’s thermal and mechanical properties as a textile raw material. FTIR analysis was used to determine the functional groups of ramie fiber before and after impregnation. Figure 6 shows the FTIR results of ramie fiber following 30, 60, and 90 min of impregnation. Before the impregnation, ramie fiber had six main peaks. The components of ramie fibers such as cellulose, hemicellulose, lignin, and water adsorbed on the fiber surface cause O–H stretching at wavenumbers 3400–3300 cm^−1^ [66,67]. The C–H stretching vibration and C=O stretching vibration of the ester/carboxylate group of hemicellulose component was indicated by absorption peaks at wavenumbers of 2897 cm^−1^ and 1740 cm^−1^ [68]. The peak at wavenumbers 1607 cm^−1^, 1424 cm^−1^, and 1024 cm^−1^ were attributed to the C–O stretching of the aromatic ring of lignin, C–H deformation of carbohydrates, and C–O and O–H vibrations in cellulose, respectively [69].

After impregnation, the functional groups of ramie fiber were evaluated using FTIR. The absorption bands tended to be the same for each treatment with different intensities, indicating that the impregnation of ramie fiber with LPU was effective. The same O–H group as ramie fiber before impregnation is shown by the peak at wavenumber 3330 cm^−1^. The peak at 2897 cm^−1^ changed to 2910 cm^−1^ after impregnation, indicating the existence of N–H groups in the ramie fiber. The wavenumber 1740 cm^−1^ was not identified, but a peak at 1600 cm^−1^ was detected, indicating the formation of C=O of urethane from LPU resins [23]. The C–N groups from primary and secondary amides were shown by the wavenumber 1515–1310 cm^−1^. The C=O vibrations of LPU, C–O–C of ether bonds, and C–H stretching linked with aliphatic and aromatic groups may also be shown at wavenumbers 1200 cm^−1^ and 1050–1015 cm^−1^ [68].

**Figure 7:**
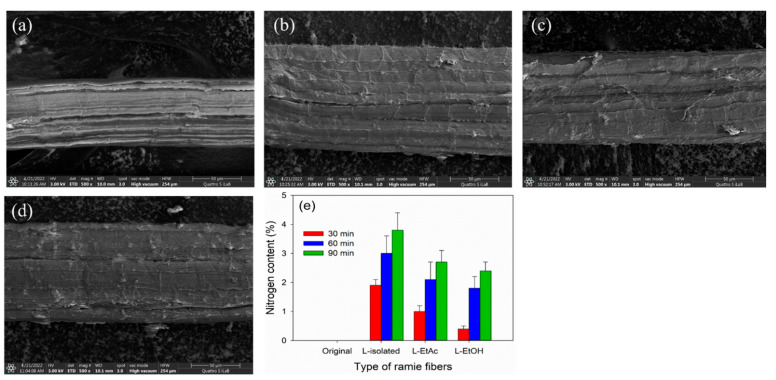
FE-SEM combined with EDX analysis of ramie fibers impregnated with lignin-based PU (LPU) resins: (**a**) original ramie fibers, (**b**) ramie–L-isolated—90 min, (**c**) ramie–L-EtAc—90 min, (**d**) ramie–L-EtOH—90 min, (**e**) nitrogen content of ramie before and after impregnation with different LPU resins for different times.

FE-SEM coupled with EDX were performed to investigate the influence of impregnation process on the surface morphology and nitrogen content of ramie fibers (Figure 7). The result showed that the original ramie fibers had more brightness color compared to the impregnated ramie fibers (Figure 7a–d). The impregnation process changed the color of the fibers to dark brown. In addition, the impregnation process covered the ramie fibers with a layer of LPU resins, which was not observed on original ramie fibers. This result was supported by the EDX analysis, revealing that the original ramie fibers contained 0% nitrogen, while the impregnated ramie fibers had a nitrogen content varying from 0.4% to 3.8% (Figure 7e). The nitrogen content increased with the longer impregnation time. The increase in nitrogen content of ramie fibers was obviously due to the LPU resins, which contained urethane groups as indicated by the results of FTIR spectroscopy (Figure 3 and Figure 6) and the weight gain (Table 5). Regardless of the impregnation time, ramie impregnated with LPU resins-L-isolated contained higher nitrogen compared to LPU resins-L-EtAc and L-EtOH. This was probably because LPU resins-L-isolated had lower viscosity than those of LPU resins-L-EtAc and L-EtOH (Table 5). This results were in line with the results of ramie impregnated with tannin-based PU resins for different impregnation times [14].

**Figure 8:**
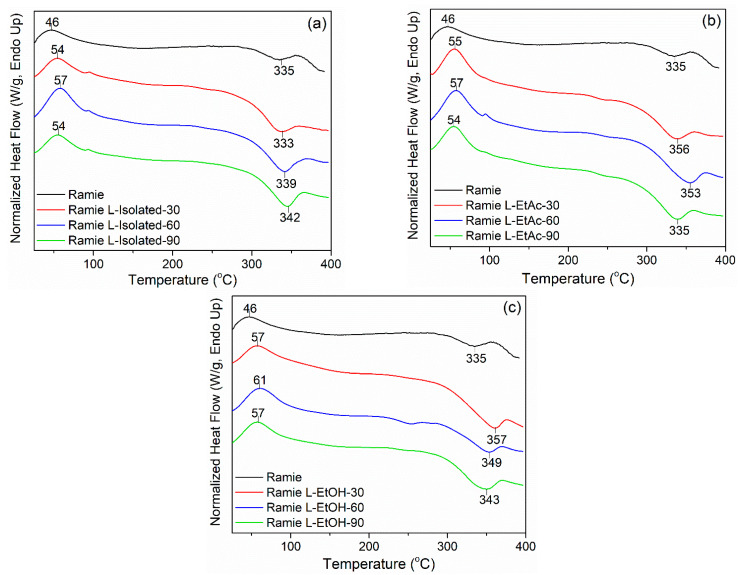
DSC analysis of before and after impregnated ramie fiber with lignin-based bio-PU: (**a**) ramie impregnated with L-Isolated, (**b**) ramie impregnated with L-EtAc, (**c**) ramie impregnated with L-EtOH.

DSC analysis was performed to evaluate the thermal properties of ramie fibers. The result showed two reaction peaks in the ramie fiber before and after impregnation (Figure 8). The first reaction peak (*Tp_1_*) is an endothermic reaction, which occurs whenever the fiber absorbs heat. The *Tp_1_* of the endothermic reaction of the original ramie fiber was 46 °C, while the *Tp_1_* of ramie fiber after impregnation was 54–61 °C. The evaporation of water contained in the fiber causes this endothermic process. The difference in *Tp_1_* in ramie fiber after impregnation was affected by the type of LPU and the impregnation time. The second reaction peak (*Tp_2_*) represents the exothermic reaction that occurs in ramie fiber. The *Tp_2_* was observed at a temperature range of 300–360 °C. The exothermic reaction is a heat-release reaction that explains the development of solid residues due to hemicellulose and lignin degradation [70]. The increase in *Tp_2_* value means that the fibers have improved high-temperature properties. The *Tp_2_* occurred at 335 °C in the original ramie fiber, while the ramie impregnated with LPU L-isolated has *Tp_2_* of 333–342 °C, ramie L-EtAc has *Tp_2_* of 335–356 °C, and ramie L-EtOH has *Tp_2_* of 343–357 °C. In this case, the impregnation time had no remarkable effect on the thermal properties of the fiber.

**Figure 9:**
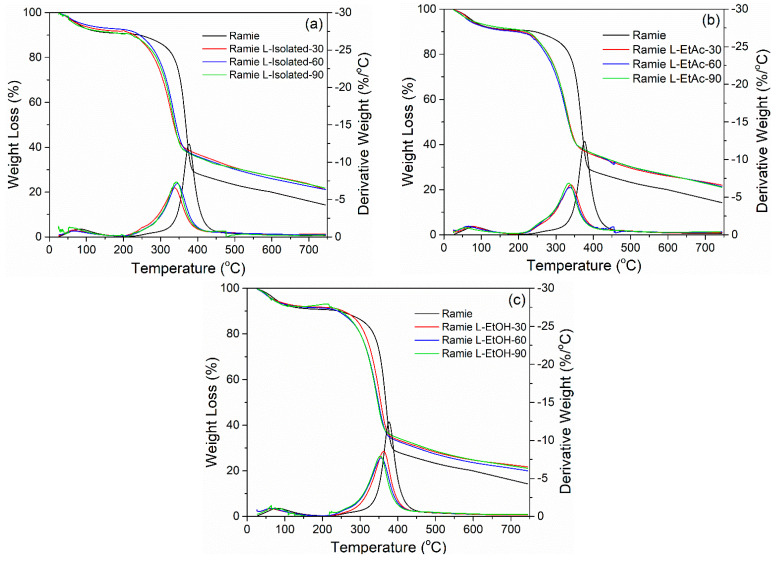
TGA—DTG of before and after impregnation of ramie fiber with lignin-based bio-PU: (**a**) ramie impregnated with L-Isolated, (**b**) ramie impregnated with L-EtAc, (**c**) ramie impregnated with L-EtOH.

Figure 9 presents the processes of thermal degradation of ramie fiber before and after impregnation with LPU resins. There are two stages of fiber degradation, including in the temperature ranges of 50–150 °C and 250–450 °C. The first step of degradation is removing water from the fiber at temperature ranges of 50–150 °C. The maximum of this stage typically appears when the heating temperature gets to 100 °C, showing a small derivative weight loss of 1–2% °C^−1^, then followed by a stable temperature with no significant fluctuations. At temperature ranges of 200–500 °C, a degradation of lignocellulosic components of the fiber occurred, including degradation of lignin, hemicellulose, and cellulose constituents [71].

At temperatures of 250–300 °C, there was a substantial loss of weight due to hemicellulose degradation, followed by an extreme loss of weight at temperatures of 300–500 °C, indicating that the cellulose and lignin components had decomposed [72]. At a temperature of 350–400 °C, maximum decomposition occurs, and lignin degradation in ramie fiber is mentioned as the limit for determining thermal stability [73]. Before impregnation, ramie fiber had a higher derivative weight of 13.0% °C^−1^, while after impregnation the derivative weight was 5–7% °C^−1^. However, the impregnated fibers began to break down earlier than original ones, namely at around 340 °C for impregnated ramie fibers and at around 370 °C for the original ramie fiber. After heating up to 700 °C, the original ramie residue was 14.3%, while the ramie residue after impregnation was 19.0–22.0% for all types of LPU and impregnation times. According to the results, the variation in impregnation time did not influence the thermal properties of the ramie fibers. The slightest weight decrease was found in ramie fiber impregnated with LPU L-EtAc for 30 min, with a weight loss of 78.0% and a residual weight of 22.0%.

**Figure 10:**
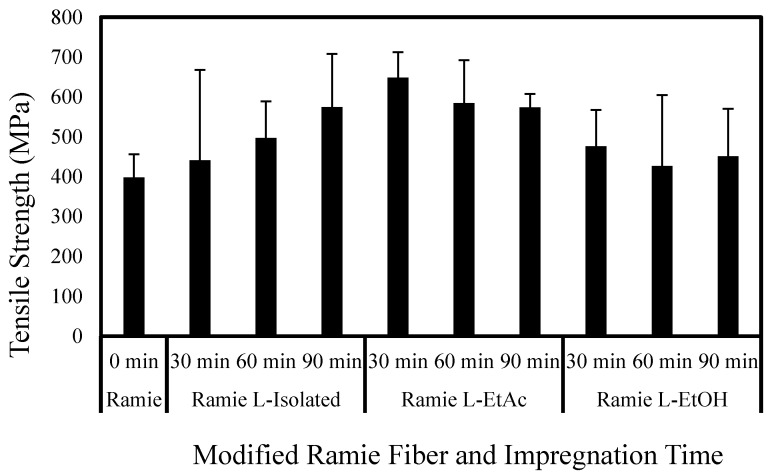
Variation of tensile strength of modified ramie fiber.

**Figure 11:**
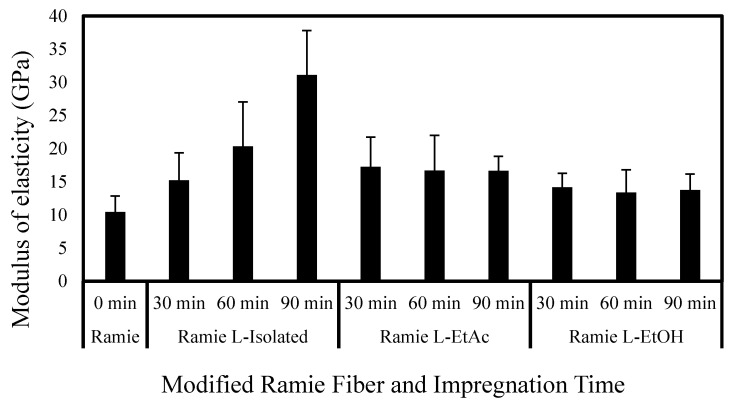
Variation of the modulus of elasticity (MOE) of modified ramie fiber.

Figure 10 and Figure 11 reveal that ramie fiber impregnated with LPU had higher fiber tensile strength and MOE values than the original ramie fibers. The presence of a phenolic hydroxyl groups in the lignin can contribute to creating a PU chain. Crosslinking between the phenolic hydroxy groups (–OH) and isocyanate groups (–NCO) enhances the degree of crosslinking and chain linkage in LPU resins. The mechanical strength of ramie fibers impregnated with LPU resins is thought to be enhanced due to these issues. The chemical composition of fiber and its internal structure and morphological characteristics influence its mechanical properties, such as tensile strength. Ramie fibers has a high microfibril angle and much cellulose, so it has an excellent tensile strength [69].

Ramie fibers exhibited improved tensile strength and MOE compared with the control after impregnation treatment. It can be seen that ramie fiber treated with LPU L-EtAc for 30 min has the highest tensile strength of 648.7 MPa. Meanwhile, ramie fiber treated with LPU L-isolated gives the highest MOE of 31.1 GPa. Generally, LPU L-EtAc treatment resulted in fibers with higher tensile strength that that of other LPU treatments. The impregnation time had no significant effect on the tensile strength of the fibers, as demonstrated by the inconsistent trend in Figure 8. The influence of impregnation time, on the other hand, is more visible in MOE, since the MOE of ramie fiber increases as the impregnation time increases. Ramie fiber treated with LPU L-EtOH has relatively poorer tensile strength and MOE compared to that of LPU L-EtAc and LPU L-isolated. This might be due to the fact that L-EtOH has the lowest phenolic hydroxyl group (7.2 mmol·g^−1^) compared to that of L-EtAc (8.1 mmol·g^−1^) and L-isolated (7.9 mmol·g^−1^) in the present study. Higher amounts of phenolic hydroxyl groups in the lignin structure may boost hydrogen bond formation between lignin and other components with numerous -OH groups, hence reinforcing the network structure and resulting in improved mechanical properties [74,75].

## 4. Conclusions

This study established the feasibility of extracting lignin from black liquor and using it as a pre-polymer in the synthesis of LPU for ramie fiber impregnation. Isolated lignin and lignin fractionation in EtAc and EtOH solvents have a relatively similar chemical structures with L-Standard. L-Standard has higher content of aliphatic O–H group and ether while L-Isolated, L-EtAc, and L-EtOH have an asymmetric OCH_3_ group and conjugate acid that did not exist in L-Standard. L-EtOH has a much higher yield but lower phenolic hydroxyl group than L-EtAc. In term of thermal characteristics, the thermal stability of L-Isolated is exceptional when comparing with L-Standard, indicated by its higher combustion residues. L-EtOH has a comparable but slightly lower thermal stability than L-Standard, while L-EtAc showed the poorest thermal stability.

LPU resins were successfully produced when L-Isolated, L-EtAc, and L-EtOH were reacted with pMDI. The resulted LPU resins displayed good thermal stability; LPU derived from L-EtOH having the highest thermal stability, followed by L-Isolated, and L-EtAc. Owing to the difference in viscosity, ramie fibers impregnated with these LPU resins exhibited different extents of weight gain. In addition, the weight gain increased along with increasing impregnation time. However, the impregnation time had no remarkable effect on the thermal properties of the impregnated fibers. The thermal stability of ramie fibers after impregnation with LPU resins was improved. The thermal stability of the ramie fibers impregnated with LPUs derived from L-Isolated, L-EtAc, and L-EtOH was more or less the same, but those fibers impregnated with LPU L-EtAc showed slightly better thermal stability. Apart from that, the tensile strength and MOE values of ramie fibers after impregnation were higher than the tensile strength and MOE values of the original ramie fibers. This study found that impregnation with LPU improved the thermal and mechanical performance of ramie fibers, allowing them to be used in a wider range of industrial applications in the future.

## Figures and Tables

**Table 1:** Characteristics of isolated lignin.

Parameters	Value	References
Yield (%)	35.9 ± 1.8	20–40 [1]
MC (%)	5.1 ± 0.7	8.1 [47]
Ash Content (%)	0.3 ± 0.02	0.5–4.3 [48]
AIL (%)	82.5 ± 1.0	85.1–91.2 [48]
ASL (%)	12.8 ± 0.7	2.1–6.0 [48]
Purity Levels (%)	95.3 ± 0.6	83–97 [48]

**Table 2:** Yield and total OH phenolic of fractionated lignin.

Type of Lignin	The Yield of Fractionated Lignin (%)	The Total Phenolic Hydroxyl Group (mmol·g^−1^)
L-Standard	-	8.1
L-Isolated	-	7.9
L-EtAc	28.9	8.1
L-EtOH	73.5	7.2

**Table 3:** FTIR band assignment of isolated and fractionated lignin.

Wavenumber (cm^−1^)				References [50,51,52,53,54]	Functional Groups
L-Standard	L-Isolated	L-EtAc	L-EtOH		
3355	3347	3374	3380	3550–3200	O-H stretching (alcohol)
2936	2917	2917	2917	2940–2820	C-H stretching (CH_3_ and CH_2_)
-	2849	2849	2849	2850–2840	C-H stretching (asymmetric OCH_3_ group)
-	1720	1720	1720	1720–1680	C=O stretching (conjugate acid)
1593	1598	1601	1597	1595	Aromatic skeletal vibrations, C=O stretching (conjugate)
1511	1513	1513	1514	1515–1505	C-C stretching (aromatic ring)
1214	1209	1208	1210	1220	C-O(H) + C-O(Ar) (OH phenolic and ether in syringol and guaiacyl)
1125	1110	1110	1113	1115	Deformations of Ar-CH (syringol)
1080	-	-	-	1085–1030	C-O(H) + C-O(C) (OH aliphatic and ether)
1030	1031	1033	1032	1030	C-O in syringyl and guaiacyl, C-H bonding in guaiacyl

**Table 4:** Pyrolysis products of lignin assessed by Py-GCMS.

No	RT (min)	Pyrolysis Product	Origin	L-Isolated	Fractionated Lignin	
					L-EtAc	L-EtOH
1	9.50	Phenol	H	3.9	2.3	4.1
2	11.76	Phenol, 2-methyl-	H	1.2	1.0	1.4
3	12.46	Phenol, 4 methyl	H	2.7	3.4	2.9
4	12.80	Guaiacol	G	14.2	10.0	14.5
5	15.97	Guaiacol, 4-methyl	G	6.8	9.1	6.8
6	16.44	Catechol	H	4.7	3.2	4.9
7	17.30	Guaiacol, 4-ethyl	G	0.4	0.1	0.4
8	18.16	Catechol, 3-methoxy	H	7.3	6.2	6.6
9	18.30	Catechol, 4 methyl	H	1.4	1.8	0.2
10	18.51	Guaiacol, 4-ethyl	G	3.9	4.7	3.6
11	19.62	Guaiacol, 4-vinyl	G	5.6	4.9	5.7
12	20.76	Syringol	S	16.8	14.1	17.4
13	21.88	Eugenol	G	0.7	0.5	0.7
14	22.22	Isoeugenol (cis)	G	1.4	1.4	1.2
15	23.30	Syringol-4-methyl	S	6.2	9.5	6.5
16	23.45	Isoeugenol (trans)	G	3.5	2.3	3.2
17	24.49	Acetoguaiacone	G	1.8	3.3	2
18	25.30	Syringol, 4-ethyl	S	3.2	4.5	3.4
19	25.53	Guaiacylacetone	G	0.7	0.5	0.9
20	26.39	Syringol, 4-vinyl	S	5.0	4.9	5.0
21	26.95	Propioguaiacone	G	0.8	1.1	0.8
22	27.22	Syringol, 4-allyl	S	0.8	0.5	0.8
23	27.39	Homosyringaldehyde	S	0.4	0.5	0.4
24	28.04	Syringol, 4-propenyl	S	0.5	0.7	0.4
25	29.73	S-4propenyl	S	2.0	1.6	1.9
26	30.49	Acetosyringaldehide	S	2.4	4.7	2.7
27	31.24	Syringylacetone	S	0.5	0.6	0.4
28	32.54	Propiosyringone	S	0.8	1.3	0.9
LH (hydroxyphenyl)				21.2	17.9	20.2
LG (guaiacyl)				39.8	37.8	39.9
LS (syringol)				38.5	42.8	39.7
S/G ratio				0.97	1.13	0.99

**Table 5:** Weight gain of ramie fiber after impregnation with lignin-based bio-PU (LPU) resins at different impregnation times.

LPU Resins	Viscosity (mPa s^−1^)	Weight Gain of Ramie Fiber (%)		
		30 min	60 min	90 min
L-Isolated	77.0	12.4 ± 1.7	15.2 ± 0.3	15.9 ± 2.4
L-EtAc	206.6	8.6 ± 1.6	9.2 ± 3.5	10.9 ± 2.6
L-EtOH	316.9	6.2 ± 0.1	6.7 ± 0.4	7.5 ± 0.7

## Data Availability

The data presented in this study are available on request from the corresponding author.
